# Impact of virtual patient engagement on glycemic control in type 2 diabetes: a retrospective observational study from GluCare hybrid care model

**DOI:** 10.3389/fendo.2025.1695381

**Published:** 2025-11-20

**Authors:** Hala Zakaria, Geethu Paul, Joelle Debs, Juman Ali, Adam Almarzooqi, Ali Hashemi, Ihsan Almarzooqi

**Affiliations:** GluCare.Health, Dubai, United Arab Emirates

**Keywords:** virtual patient engagement, glycemic control, type 2 diabetes, hybrid care model, HbA1c, digital health, remote monitoring

## Abstract

**Background:**

Type 2 diabetes mellitus (T2DM), a chronic metabolic disorder requiring sustained glycemic control to prevent complications. Traditional in-clinic care often limits patient engagement to periodic visits, leaving gaps in continuous diabetes management. Digital health interventions, including virtual two way patient engagement between patients and the care team may enhance adherence and outcomes. However, the impact of different engagement modalities on glycemic control remains underexplored. This study evaluates the impact of virtual patient engagement within the GluCare hybrid care model on glycemic control in individuals with T2DM.

**Methods:**

This retrospective observational study included T2DM patients(n=125) enrolled in GluCare’s hybrid care program. Participants were stratified into two groups based on glycemic control: controlled (HbA1c <7%) and poorly controlled (HbA1c ≥7%). Patient engagement was categorized into inbound (patient-initiated) and outbound (provider-initiated) interactions. Clinical and metabolic parameters, including HbA1c, blood pressure, lipid profile, inflammatory markers, renal function, and anthropometric measures, were assessed at baseline and 12 months.

**Results:**

Participants in the poorly controlled group exhibited a mean HbA1c reduction of −2.4% (p < 0.001), while controlled patients improved −0.3% (p < 0.001). This magnitude exceeds the −0.3% to −0.5% typically reported for digital-only interventions, indicating clinically meaningful improvement. The higher number of virtual interactions was associated with improved glycemic control (β = –0.007; 95% CI: –0.011 to –0.002; p = 0.003) and remained significant after adjusting for age, BMI, medication use, and glycemic control group (β = –0.006; 95% CI: –0.010 to –0.002; p = 0.001). Both controlled and poorly controlled groups achieved similar HbA1c levels at 12 months (p = 0.205). Engagement peaked at 3 months and declined thereafter, with outbound interactions consistently exceeding inbound ones However, Poorly controlled group more prescribed on GIP/GLP-1 receptor agonists (59.6%), insulin (75%) and statin therapy (56.8%) compared to the controlled group.

**Conclusion:**

Virtual patient engagement, particularly provider-initiated interactions, plays a crucial role in optimizing glycemic control and improving metabolic outcomes in T2DM. Hybrid care models that integrate continuous remote monitoring with periodic in-clinic visits offer a viable approach to sustaining patient adherence.

## 1 Introduction

Type 2 diabetes mellitus (T2DM) is a chronic and prevalent metabolic disorder characterized by insulin resistance, impaired insulin secretion, and hyperglycemia, affecting millions worldwide (1, 2). It is associated with an increased risk of cardiovascular, renal, and neuropathic complications, contributing to significant morbidity and mortality (3, 4). Effective glycemic control, typically measured by glycated hemoglobin (HbA1c), is essential to reduce these risks and improve long-term outcomes (5). However, achieving and maintaining optimal glycemic control remains a challenge for many patients, despite advancements in pharmacological treatments, access to GLP-1 medications and lifestyle interventions (6).

Patient engagement is a critical component in diabetes management, encompassing active participation in healthcare decisions, adherence to treatment regimens, and consistent communication with healthcare providers (7). The current episodic nature of patient engagement, usually done on a quarterly basis in a physical setting, is usually limited to a few minutes’ discussion with a care team mostly around pharmacotherapy. Digital health interventions, including remote monitoring, mobile applications, and patient portals, have demonstrated potential in enhancing engagement and improving clinical outcomes (8). Studies have shown that patients who regularly engage with digital platforms exhibit better glycemic control, reduced healthcare utilization, and improved quality of life (9, 10). Systematic reviews and meta-analyses of digital health interventions have consistently shown modest reductions in HbA1c, typically in the range of –0.3% to –0.5%, with some variability depending on intervention intensity and population (11, 12). Several digital solutions demonstrate limited durability or modest efficacy rather than outright failure. Their lower impact often stems from limited integration with clinical teams, fragmented feedback loops, and absence of behavioral reinforcement mechanisms. This disconnection from coordinated medical care restricts providers’ ability to intervene early and sustain patient motivation. These findings, though promising, suggest that digital-only models often achieve limited impact in glycemic control when disconnected from the patient’s broader clinical care pathway. Moreover, healthcare providers play a crucial role in fostering engagement through timely feedback, personalized communication, and proactive support (13). In addition, the entire lifestyle modification and behavioral change element is left to patients with minimal involvement from providers.

Understanding the relationship between different engagement patterns, such as inbound (patient-initiated) and outbound (provider-initiated) interactions, is essential for optimizing digital health interventions and tailoring support to patients’ needs. While digital-only solutions provide convenience and accessibility, they may lack the essential patient-provider relationship needed for sustained engagement and adherence. Many digital solutions have largely failed as the teams managing patients virtually are disconnected from traditional providers who manage patients in the clinical setting (14). Thus, hybrid models like the GluCare.Health approach may outperform digital-only programs by embedding continuous remote monitoring within a clinical environment that provides personalized feedback, timely medication adjustment, and multidisciplinary behavioral coaching. The effectiveness of this hybrid model has been highlighted in the NEJM Catalyst *Innovations in Care Delivery Models* paper, demonstrating its role in improving patient engagement and clinical outcomes in diabetes management (15). Additionally, previous research conducted within our institution has provided further evidence supporting the benefits of a hybrid approach in sustaining long-term engagement and optimizing metabolic control (16, 17). This dual approach enhances patient engagement, ultimately leading to improved adherence and clinical outcomes. Previous research has suggested that frequent interactions, particularly outbound communications, are associated with improved adherence and glycemic outcomes (18, 19). However, the specific impact of these interaction types on glycemic control remains underexplored. This study seeks to address the important evidence gap by quantifying how virtual engagement intensity relate to glycemic improvement in a real-world hybrid program.

The study aims to evaluate the effect of total virtual engagement (both provider- and participant-initiated interactions), on changes in HbA1c over 12 months and to compare the engagement patterns between participants with controlled and poorly controlled diabetic group. Also as exploratory to observe the concurrent changes in the clinical parameters between the groups from baseline to 12 months.

## 2 Methodology

### 2.1 Settings and description of GluCare’s hybrid model

The GluCare hybrid model integrates traditional in-clinic, patient-centered care with a data-driven Remote Continuous Data Monitoring (RCDM) approach to enhance T2DM management (15). This model aims to improve patient engagement and optimize metabolic control by combining real-time monitoring with direct interaction from a multidisciplinary care team and uses the basis that higher engagement can be obtained when centered around the patient’s own biomarkers rather than generic education. Patients receive individualized consultations from a team consisting of physicians, dietitians, diabetes educators, exercise practitioners and health coaches. During physical quarterly visits, comprehensive assessments focus on medication adherence, metabolic parameters, lifestyle modifications, and engagement with digital tools. Personalized treatment plans are reinforced through real-time feedback and digital integration. The RCDM component utilizes connected health technologies to track patient adherence and metabolic trends, allowing healthcare teams to intervene when needed. Patients log meals, symptoms, and lifestyle data through the GluCare mobile application. Connected devices including Continuous Glucose Monitors (CGM) and ŌURA Rings record glucose, heart-rate variability, sleep, and activity in real time. All components of the program are offered uniformly to every patient as part of standard care; however, patients may choose to opt out of any element at their discretion. The care team reviews incoming data daily and provides feedback or intervention as clinically indicated. Outbound (provider-initiated) and inbound (patient-initiated) interactions constitute the virtual-engagement metric analyzed in this study. Outbound contact follows a structured protocol: at least weekly during the first three months, bi-weekly from months 4 to 6, and thereafter at clinician discretion depending on biometric trends and engagement level. This proactive cadence ensures timely medication titration, reinforcement of lifestyle goals, and sustained accountability. The GluCare model represents a novel and highly structured care delivery setting that differs from typical diabetes care in many regions, where follow-up is often limited to general practitioner visits and access to technologies such as CGMs or digital coaching may be restricted. At GluCare Clinic, patients are routinely offered continuous glucose monitoring and access to wearable devices as part of standard care.

### 2.2 Study design and participants

This retrospective observational study was conducted at GluCare Integrated Diabetes Center, Dubai, UAE, involving adult patients diagnosed with T2DM who were enrolled in a hybrid diabetes management program. A total of 125 participants were classified into two groups based on their glycemic control over a 12-month period: controlled (HbA1c < 7%) and poorly controlled (HbA1c ≥ 7%).

#### 2.2.1 Inclusion and exclusion criteria

Medical records of adult patients (aged ≥18 years) with a confirmed diagnosis of T2DM who were enrolled in the GluCare.Health hybrid model program between January 2021 and October 2024 were reviewed. Patients with only a baseline HbA1c measurement and no follow-up data were excluded. From the remaining cohort, individuals with complete clinical and engagement data at baseline and at 12 months were included in the analysis. All included patients completed a 12-month follow-up. This retrospective selection ensured data completeness for longitudinal outcome analysis. Inclusion criteria were as follows: (1) adults aged 18 years or older; (2) a confirmed diagnosis of T2DM; (3) participation in the Remote Chronic Disease Management (RCDM) program for at least 12 months; and (4) availability of HbA1c measurements at both baseline and 12 months. Exclusion criteria included: (1) a diagnosis of type 1 diabetes; (2) pregnancy during the study period; (3) withdrawal from the RCDM program; and (4) incomplete clinical or engagement data.

### 2.3 Data collection

Data were extracted from electronic medical records, including demographic information, clinical parameters, medication profiles, and engagement metrics. Clinical parameters included glycated hemoglobin (HbA1c), systolic and diastolic blood pressure, cholesterol levels (total cholesterol, LDL, HDL, and triglycerides), renal function markers (eGFR and creatinine), inflammatory markers (hs-CRP), liver function tests (AST and ALT), uric acid, urinary microalbumin, weight, height, and waist circumference. Medication usage was recorded, including metformin, GLP-1 receptor agonists, GIP/GLP-1 receptor agonists, insulin (long-acting and short-acting), SGLT2 inhibitors, DPP-4 inhibitors, sulfonylureas, thiazolidinediones, Oral semaglutide, and acarbose, as well as antihypertensive medications such as ACE inhibitors, beta-blockers, angiotensin II receptor antagonists, calcium channel blockers, and statin therapy. Engagement metrics were divided into: I. Inbound interactions: patient-initiated messages, uploaded logs, or queries regarding medications, symptoms, or self-management, and II. outbound interactions: provider-initiated contacts including check-ins, feedback, medication changes, motivational or educational messaging, and appointment reminders. Each message exchanged between the patient and the care team was counted as one interaction, regardless of direction. Routine non-substantive exchanges—such as greetings or thank-you responses, were excluded to ensure only clinically relevant communications were analyzed. Interaction counts were recorded at 3-month intervals (0–3, 3–6, 6–9, and 9–12 months) and analyzed both cumulatively and by interval.

### 2.4 Outcome measures

The primary outcome of the study was the change in HbA1c levels over a 12-month period, stratified by baseline glycemic control status (controlled vs. poorly controlled). Exploratory outcomes included changes in clinical and biochemical parameters, such as blood pressure (systolic and diastolic), lipid profile (total cholesterol, LDL, HDL, and triglycerides), inflammatory markers (hs-CRP), liver function tests (AST and ALT), renal function markers (eGFR and creatinine), urinary microalbumin, uric acid levels, and anthropometric measures (BMI, weight, and waist circumference), also assessed between the two glycemic groups over the same period.

### 2.5 Ethical approval

Ethical approval was obtained from the Dubai Health Authority (DHA) (Ethical approval number DSREC-03/2025_30), and patient data were anonymized to maintain confidentiality. Written informed consent was obtained from all patients during their initial visit.

### 2.6 Statistical analysis

Baseline characteristics of the study participants were summarized using descriptive statistics. Categorical variables were presented as frequencies and percentages, while continuous variables were reported as means with standard deviations (SD) for normally distributed data or as medians with interquartile range (IQR) for non-normally distributed data. Differences in baseline characteristics between controlled and poorly controlled groups were assessed using chi-square or Fisher’s exact tests for categorical variables and independent t-tests for continuous variables. Changes in the HbA1c levels at 12 months between the two groups were compared using analysis of covariance (ANCOVA) model adjusted for the baseline HbA1c level and the total number of virtual interactions. The changes in the engagement levels over time were assessed using repeated measures ANOVA separately for both inbound and outbound interactions. The complete case analysis of paired data was used to assess the changes in clinical parameters between baseline and the 12-month follow-up using paired t-tests or Wilcoxon signed-rank tests as appropriate, separately for both the controlled and poorly controlled group. Engagement levels were stratified into different categories based on the quartiles of the total interactions to allow for meaningful group comparisons. Differences in clinical outcomes across engagement levels were examined using the Kruskal-Wallis test. Spearman’s rank correlation was used to assess the correlation between changes in HbA1c levels and the total number of interactions. All statistical analyses were conducted using R version 4.4.0, with a significance level set at p-value < 0.05.

## 3 Results

### 3.1 Basic demographics and characteristics

Baseline characteristics between controlled (n=63) and poorly controlled (n=62) groups were compared, the age and the gender distribution were similar between the two groups (**Table 1**). However, differences were observed in medication profiles, with participants in the poorly controlled group more likely to be on GIP/GLP-1 receptor agonists (59.6%), insulin therapy (75%) and statin therapy (56.8%) compared to the controlled group. No significant differences were noted in hypertension treatment, including the use of ACE inhibitors, beta-blockers, or angiotensin II receptor antagonists. Median total inbound engagement in the portal during the study period was comparable between the groups (p =0.92).

**Table 1:** Baseline characteristics of controlled and poorly controlled T2DM patients (n =125).

Characteristics	Controlled (n =63)	Poorly controlled (n=62)	P value
	Mean ± SD/Median (IQR)/n (%)		
Age	47.9 ± 10.9	47.5 ± 8.7	0.81
Gender			
Male	50 (50.5)	49 (49.5)	0.96
Female	13 (50)	13(50)	
BMI (Mean ± SD)	30.12 ± 5.48	30.29 ± 6.08	0.88
Waist circumference (Mean ± SD)	101.65 ± 12.67	104.31 ± 14.8	0.32
Medication Profile (n (%))			
Diabetes Treatment			
GIP/GLP-1	23 (40.4)	34 (59.6)	0.04*
GLP-1	11 (39.3)	17 (60.7)	0.18
Oral Hypoglycemic Agents	17 (60.7)	11 (39.3)	0.22
Insulin	4 (25)	12 (75)	0.03*
Hypertension Treatment			
BP medication			
ACE Inhibitors	2 (66.7)	1 (33.3)	0.99
Beta Blockers	1 (33.3)	2 (66.7)	0.62
Angiotensin II receptor antagonist	6 (37.5)	10 (62.5)	0.3
Statin Therapy	35 (43.2)	46 (56.8)	0.03*
Total inbound engagement	24 (12,41)	28 (9,47)	0.92

*P value <0.05 shows statistical significance from Chi-square test.

### 3.2 Effect of virtual patient engagement on glycemic control at 12 months

Both the groups demonstrated a reduction in HbA1c over time, with the HbA1c at 12 months being 6.59% (95% CI: 6.29–6.89) in the poorly controlled group and 6.26% (95% CI: 5.96–6.56) in the controlled group. Also, there was no statistically significant difference in glycemic control at 12 months between the two groups (P = 0.205) adjusted for their baseline HbA1c level and the total number of virtual interactions. A higher number of virtual interactions was associated with improved glycemic control (β = –0.007; 95% CI: –0.011 to –0.002; p = 0.003). This association remained significant after adjusting for age, BMI, medication use, and glycemic control group (β = –0.006; 95% CI: –0.010 to –0.002; p = 0.001).

### 3.3 Improvements in clinical and anthropometric parameters at baseline versus 12 months among controlled and poorly controlled groups

Blood pressure also improved in both groups, with systolic BP decreasing from 123.22 mmHg to 119.02 in the controlled group and from 128.2 mmHg to 118.1mmHg (*p* < 0.001) in the poorly controlled group. Similarly, diastolic BP declined significantly in both groups (*p* < 0.001) (**Table 2**). Lipid profile improvements were evident, particularly in the poorly controlled group, where total cholesterol and LDL dropped significantly (*p* < 0.001). HDL levels significantly improved in both groups (*p* < 0.001). Triglyceride levels also declined significantly in both groups (*p* = 0.033 and *p* = 0.001, respectively). Inflammatory markers, such as CRP, significantly decreased in both groups (*p* < 0.001), while liver function parameters, including AST and ALT, showed a significant reduction only in the poorly controlled group (*p* < 0.001). Uric acid levels decreased significantly in the controlled group (*p* = 0.001) but remained unchanged in the poorly controlled group (*p* = 0.266). Urinary microalbumin levels remained stable in the controlled group (*p* = 0.111) but showed a significant reduction in the poorly controlled group (*p* < 0.001). Anthropometric parameters improved consistently across both groups. BMI significantly decreased by ~ 2 kg/m ^2^ in both the controlled and poorly controlled group (*p* < 0.001). Similarly, waist circumference reduced significantly in both groups (*p* < 0.001).

**Table 2:** Clinical and anthropometric parameters at baseline vs 12 months among controlled and poorly controlled T2DM patients (n =125).

Characteristics	Controlled (n =63)			Poorly controlled (n=62)		
	At baseline	At 12 months	P value	At baseline	At 12 months	P value
Blood Pressure						
Systolic BP	123.22 ± 12.95	119.02 ± 12.84	**0.028** ^a^	128.2 ± 14.1	118.1 ± 12	**<0.001** ^a^
Diastolic BP	78.75 ± 8.43	74.32 ± 6.97	**<0.001** ^a^	81 ± 8.2	75.5 ± 7.1	**<0.001** ^a^
Lipid Profile						
Cholesterol	160.4 ± 44.29	150.42 ± 39.89	0.067	185.6 ± 48	154.4 ± 43.2	**<0.001** ^a^
LDL-C	99.65(71.95,133.65)	83.75(62.03,111.63)	**0.022** ^b^	120(92.9,146.1)	81.3(61.9,116.1)	**<0.001** ^b^
HDL-C	44.5(36.6,51.7)	46.3(39.6,56.7)	**<0.001** ^b^	42.2(37.1,47.9)	43.1(38.4,52.6)	**0.001** ^b^
Triglycerides	137.3(94.1,174.1)	103.5(81.3,152.6)	**0.033** ^b^	168.4(131.5,238.5)	146.8(99.1,207.6)	**0.001** ^b^
Inflammatory markers						
hs-CRP	0.2(0.1,0.4)	0.1(0.1,0.3)	**<0.001** ^b^	0.2(0.1,0.5)	0.1(0.1,0.3)	**<0.001** ^b^
Liver function parameters						
AST	19(16.5,24.6)	21.5(17,24.5)	0.359	23.9(18.9,29)	19.4(16.4,23.9)	**<0.001** ^b^
ALT	25.1(19.3,37.4)	22.6(17,31.4)	0.175	37(24.5,47)	25.1(18.5,30.3)	**<0.001** ^b^
eGFR	110.5 ± 27.7	107.6 ± 30	0.106	112.6 ± 35.7	104.6 ± 26.8	**0.027** ^a^
Uric Acid	5.8 ± 1.6	5.1 ± 1.6	**0.00**1^a^	5.4 ± 1.4	5.2 ± 1.4	0.266
Urinary Microalbumin	5.7(4.6,19.2)	6.2(4.9,16)	0.111	7.7(5.3,21.6)	5.8(4.6,8.4)	**<0.001** ^b^
Anthropometric measures						
BMI	30.1 ± 5.5	28 ± 4.5	**<0.001** ^a^	30.3 ± 6.1	28.3 ± 5.4	**<0.001** ^a^
Waist circumference	101.7 ± 12.7	96.5 ± 10.6	**<0.001** ^a^	104.3 ± 14.8	98.2 ± 13.5	**<0.001** ^a^

*P value < 0.05 shows statistical significance from ^a^ paired t-test and ^b^ Wilcoxon signed rank test.

Proportions of patients prescribed GLP-1 and GIP/GLP-1 receptor agonists were similar at baseline and 12 months in both groups. In the poorly controlled group, GIP/GLP-1 use increased slightly from 54.8% to 56.5%, while in the controlled group it increased from 36.5% to 39.7%. GLP-1 use slightly declined in both groups over the same period. (**Supplementary Table S2**).

### 3.4 Patient engagement among controlled and poorly controlled group

**Figure 1:**
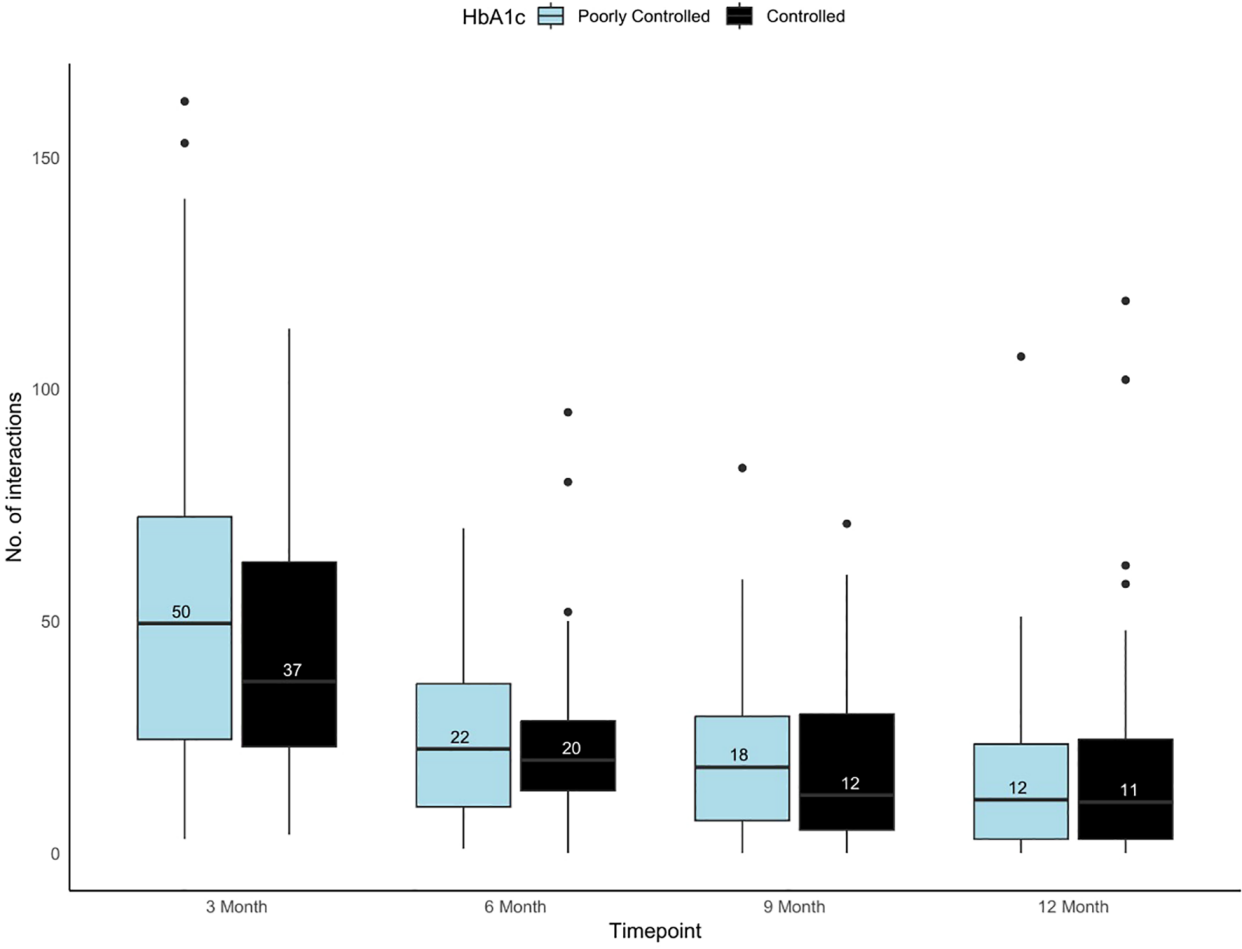
Box and whisker plot showing the variability in the total number of virtual interactions in portal among controlled and poorly controlled groups across timepoints. Black box plot represents the controlled and light blue box plot represents the poorly controlled group.

The distribution of the total virtual interactions over time among controlled and poorly controlled groups were presented in **Figure 1**. Over time, both inbound and outbound interactions, shows a significant decline from 3 months to 12 months in controlled and poorly controlled group (p<0.001). At the 3-month mark, both groups exhibited the highest number of interactions, for the poorly controlled and controlled group. The IQR also narrowed as time progressed, indicating reduced variability in interactions over time (**Figure 1**). In both the groups, outbound interactions (provider-initiated) were more frequent than inbound interactions (patient-initiated) across all time points. Also, no significant difference was observed between the groups with respect to the inbound and the outbound interactions over time. (**Supplementary Table S1**).

**Figure 2:**
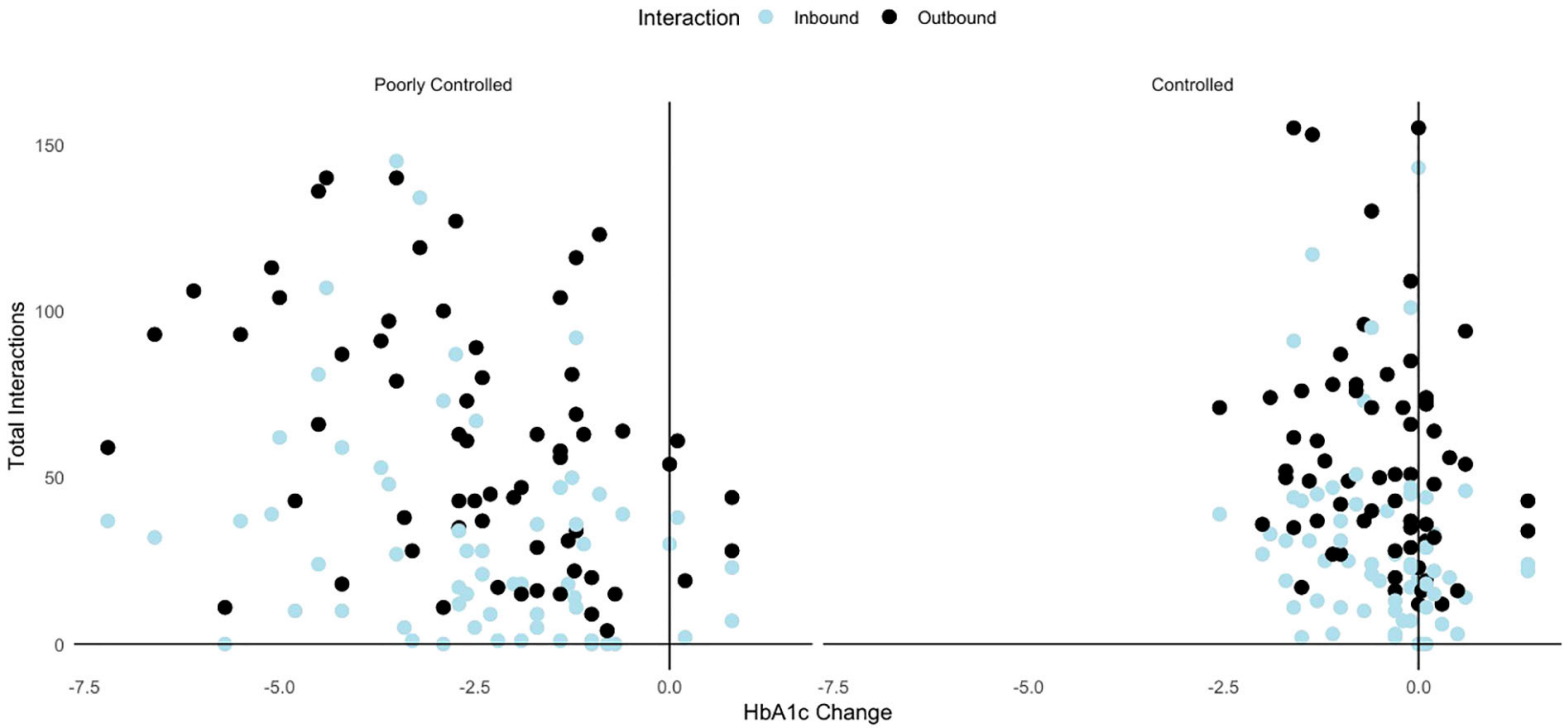
Scatterplot showing correlation between inbound interactions and outbound interactions and the change in HbA1c levels among controlled and poorly controlled groups.

Correlation analysis shows that patients with greater reductions in HbA1c demonstrated higher interaction counts, particularly in poorly controlled group, the change in HbA1c levels is positively correlated with total inbound interactions (r= 0.288, p =0.023) and outbound interactions (r = 0.385, p = 0.002). Similarly in the controlled group, the correlations were weaker for inbound interactions (r = 0.246, p = 0.052) and outbound interactions (r = 0.200, p = 0.107) though statistically insignificant (**Figure 2**).

### 3.5 Association of virtual interaction levels with clinical outcomes

Patients were divided into four groups based on their total number of interactions (<45, 45–75, 75–120, and >120). Higher interaction levels were associated with greater reductions in HbA1c, with the most significant decrease observed in the >120 interactions group (-1.6 [-3.9, 0.8], p = 0.02). (**Table 3**) Reductions in BMI and waist circumference were directionally greater in groups with higher interactions, though these differences are not statistically significant (p = 0.586 and p= 0.057 respectively).

**Table 3:** Virtual interaction levels and glycemic and anthropometric outcomes.

		Median (IQR)			
Virtual Interactions	**Inbound**	24 (10,44)			
	**Outbound**	52 (32,78)			
	**Total**	76 (45,119)			
Outcome measure	**Interactions**				
	**<45**	**45-75**	**75-120**	**>120**	**P value**
	**(n=32)**	**(n=29)**	**(n=33)**	**(n=31)**	
HbA1c reduction	-1 (-1.7,0)	-0.9 (-2,-0.1)	-1.3 (-1.9,-0.2)	-1.6 (-3.9,0.8)	0.020*
BMI	-1.3 (-2.3,-0.3)	-1.6 (-3.7,-0.3)	-1.7 (-2.7,-0.8)	-2.1 (-4.4,-1.1)	0.586
Waist Circumference	-3.5 (-7.4,-0.5)	-6.1 (-10.2,-3.5)	-6.6 (-9.2,-2)	-6.8 (-14,-3.5)	0.057

*P value < 0.05 shows statistical significance from Kruskal Wallis test.

## 4 Discussion

This retrospective observational study assessed the impact of a hybrid diabetes management program on glycemic control, clinical outcomes, and patient engagement patterns among adults with T2DM. Our findings indicate that patients with poorly controlled HbA1c at baseline exhibited substantial glycemic improvements over 12 months, along with significant enhancements in cardiometabolic and inflammatory markers. Notably, the poorly controlled group exhibited a more pronounced decline in HbA1c (-2.5%) compared to the controlled group (-0.5%), suggesting that individuals with higher baseline HbA1c levels may experience greater benefits from structured digital health interventions. However, given the markedly higher baseline HbA1c levels in this subgroup, part of the observed improvement may reflect natural regression toward the mean rather than intervention effects alone. While our study demonstrated a marked HbA1c reduction of 2.5% in the poorly controlled group, this magnitude exceeds those typically reported in randomized controlled trials and systematic reviews of digital health interventions. Meta-analyses have shown that digital interventions, including mobile apps and remote coaching, generally yield HbA1c reductions in the range of –0.3% to –0.5% over 6–12 months (11, 12). For instance, a 2024 systematic review by Kerr et al. reported a mean HbA1c reduction of –0.31% across 23 RCTs using digital tools for diabetes self-management, while Fadhil et al. found a mean effect size of –0.26% in prediabetic populations using mobile applications (11, 12). These findings highlight the potential advantage of hybrid models like ours, which combine continuous virtual interaction with in-person care. Nevertheless, a key factor that could have contributed to the improvement is the higher use of GIP/GLP-1 receptor agonists among poorly controlled participants (59.6% vs. 40.4%, p=0.04), as these medications independently produce HbA1c reductions of approximately 1.0–2.0%. Therefore, their contribution to glycemic improvement should be acknowledged as a significant confounder. The more substantial improvements observed in our study may be attributed to higher engagement intensity, integrated multidisciplinary support, and contextual tailoring of care within a unified clinical ecosystem.

### 4.1 Glycemic and cardiometabolic outcomes

While our study demonstrated a marked HbA1c reduction of 2.5% in the poorly controlled group, this magnitude exceeds those typically reported in randomized controlled trials and systematic reviews of digital health interventions. Importantly, beyond statistical significance, this reduction is also clinically meaningful. Evidence from the UK Prospective Diabetes Study (UKPDS 35) demonstrated that every 1% absolute reduction in HbA1c is associated with a 37% reduction in microvascular complications, a 14% reduction in myocardial infarction, and a 21% reduction in diabetes-related mortality (20). Furthermore, the Minimal Clinically Important Difference (MCID) for HbA1c has been estimated to be 0.3–0.5% (21). The magnitude of reduction observed in our study exceeds this threshold, underscoring the clinical relevance of hybrid care models in delivering tangible improvements in long-term outcomes for patients with type 2 diabetes. Beyond glycemic control, improvements in cardiometabolic parameters were evident. Systolic and diastolic blood pressures decreased significantly in both groups, aligning with previous studies indicating that digital health interventions contribute to improved cardiovascular health in T2DM patients (19). A significant reduction in inflammatory markers, such as hs-CRP, was observed across both groups, highlighting the potential anti-inflammatory benefits of glycemic optimization. Interestingly, the decline in uric acid observed only among the controlled group (p = 0.001) may reflect differences in dietary patterns or medication profiles such as urate-lowering agents. Conversely, the significant improvements in liver enzymes (AST, ALT) among the poorly controlled group likely reflect metabolic recovery secondary to improved glycemic regulation rather than direct intervention effects. Improvements in lipid profiles were particularly notable in the poorly controlled group, with substantial reductions in total cholesterol and LDL cholesterol levels. These changes likely reflected due to increased use of lipid-lowering agents such as statins and behavioral modifications (e.g., dietary changes promoted through digital health coaching). Nonetheless, because the study analyzed only completers, selection bias cannot be excluded. Patients who remained engaged for 12 months may differ in motivation, health literacy, or disease severity from those who discontinued earlier. This absence of an intention-to-treat analysis limits causal inference and may overestimate observed effects. While multiple metabolic parameters showed improvement, these findings should be interpreted with caution given the risk of Type I error across numerous secondary outcomes.

### 4.2 Patient engagement and its role in glycemic improvement

The findings of this study highlight the critical role of patient engagement in glycemic control among individuals with type 2 diabetes. Patients classified as poorly controlled exhibited significantly higher engagement levels, particularly in outbound interactions initiated by healthcare providers. This trend suggests that increased provider-led communication may be instrumental in improving glycemic outcomes, as reflected in the greater HbA1c reduction observed in the poorly controlled group. The significant decrease in HbA1c (-2.4% vs. -0.3%) in the poorly controlled group supports the hypothesis that active and frequent engagement is associated with better diabetes management. Our analysis of patient engagement patterns provides a relationship between interaction frequency and glycemic outcomes. ​Although both groups demonstrated peak engagement at the 3-month mark, interaction counts progressively declined over time, indicating challenges in maintaining long-term engagement. Several factors may contribute to this decline. One major reason is the diminished perceived need for support, as patients may feel that the initial three months provide sufficient guidance, leading them to believe they can manage independently without continuous engagement. This can result in reduced utilization of digital health tools and less frequent communication with healthcare providers. Additionally, reduced motivation over time is a known barrier, as the initial enthusiasm for structured diabetes management may wear off, leading to what is often termed “engagement fatigue,” particularly when patients do not perceive immediate benefits from continued participation. Furthermore, behavioral and psychological barriers such as stress, lifestyle changes, and competing priorities can interfere with sustained adherence. The poorly controlled group consistently exhibited higher median engagement than the controlled group, likely reflecting increased clinical support needs. Outbound interactions appeared more effective than inbound ones, likely because they represent proactive clinician follow-up triggered by biometric data, medication changes, or behavioral prompts. Importantly, when patients are more proactive in sharing data or concerns, this often elicits additional outbound responses from the care team, creating a dynamic feedback loop that further enhances engagement. Importantly, outbound interactions were more frequent than patient-initiated ones, underscoring the proactive need when using digital health interventions. Greater reductions in HbA1c along with BMI, waist circumference was observed among patients with higher engagement levels, particularly in those with >120 interactions. This supports prior research indicating that structured engagement strategies, particularly proactive provider outreach, enhance glycemic outcomes in T2DM patients (22). Although the association between total interaction count and HbA1c improvement was statistically significant (β = −0.003, 95% CI −0.005 to 0.0001, p = 0.05), the confidence interval includes zero. Therefore, the relationship should be interpreted with caution as marginal rather than definitive. Approximately 333 clinically relevant interactions across a year may be associated with a 1% HbA1c reduction, but this should not be considered a causal threshold.

Our study also highlights the importance of sustaining engagement over time. While initial engagement levels were high, a gradual decline in interactions was observed by the 12-month mark. Previous research suggests that maintaining digital engagement in chronic disease management requires continuous reinforcement strategies, such as personalized reminders, gamification, and adaptive interventions tailored to patient needs (23). Future iterations of hybrid care models should explore integrating these elements to sustain long-term engagement and adherence. To address the observed decline in engagement, future implementations of hybrid care models may benefit from incorporating behavioral reinforcement strategies that are proven to enhance digital adherence. These may include personalized nudges delivered through the app based on user behavior, gamification features that reward consistent engagement, and adaptive interventions that tailor the frequency and content of communications to each patient’s engagement profile. Additionally, automated reminders and prompts, integrated with meaningful clinical feedback, may help maintain motivation and encourage ongoing participation. Embedding such features into the digital infrastructure could mitigate engagement fatigue and support long-term adherence to both remote monitoring and in-clinic follow-up.

### 4.3 Strength and limitations

This study provides valuable insights into the impact of patient engagement on glycemic control within a hybrid care model. The integration of in-clinic and remote monitoring allowed for a comprehensive assessment of how patient-provider interactions influence metabolic outcomes. The use of objective engagement data, including inbound and outbound interactions, strengthens the reliability of findings. Additionally, the 12-month follow-up period ensures the evaluation of sustained effects on glycemic and cardiometabolic parameters. However, as a retrospective observational study, causality cannot be established between engagement levels and clinical improvements. Unlike randomized controlled trials, no intention-to-treat analysis could be applied, and only patients who completed the full 12-month follow-up were included. This introduces potential selection bias, as individuals who dropped out or disengaged may differ systematically from those retained in the analysis. Moreover, important confounders such as socioeconomic status, comorbidities, and medication adherence were not captured in this dataset, which may have influenced both engagement behavior and clinical outcomes. Diabetes duration, a clinically relevant variable known to impact glycemic trajectories and treatment response, was not consistently documented and therefore could not be included in the analysis. The reliance on portal-based engagement data may also have excluded other forms of patient-provider communication, such as informal or undocumented interactions. The subgroup analysis may lack sufficient statistical power to confirm the association. A larger, adequately powered study based on the observed interaction levels is required to establish causality. Furthermore, the decline in interactions over time suggests potential challenges in maintaining long-term engagement, warranting further research on strategies to sustain participation in digital health programs.

## 5 Conclusion

This study highlights the significant role of patient engagement in improving glycemic control and overall metabolic outcomes in individuals with type 2 diabetes. Higher engagement levels, particularly outbound interactions initiated by multidisciplinary healthcare provider team, were associated with greater reductions in HbA1c and improvements in other clinical parameters. The magnitude of HbA1c reduction (−2.4%) observed among poorly controlled participants is clinically meaningful and exceeds reductions typically reported in digital-only interventions, underscoring the added value of hybrid models. However, as this was a retrospective observational study, causality cannot be inferred, and the associations observed should be interpreted with caution. Future work should move beyond observational data to test causal relationships through pragmatic randomized trials or stepped-wedge hybrid implementation designs. These findings reinforce the importance of structured, proactive engagement strategies in hybrid diabetes care models, where continuous monitoring and personalized interventions can drive better adherence and clinical outcomes. However, the observed decline in engagement over time underscores the need for strategies to sustain patient participation in digital health programs. Future research should explore optimal engagement frequencies, behavioral reinforcement strategies, and the integration of predictive analytics to enhance long-term patient adherence and diabetes management outcomes. Beyond clinical outcomes, the cost-effectiveness and implementation feasibility of such models in diverse and resource-constrained healthcare settings warrant further investigation. Demonstrating economic sustainability and scalability will be key to informing policy and wider adoption.

## Data Availability

The raw data supporting the conclusions of this article will be made available by the authors, without undue reservation.
