# Supplementary material for: Impact of virtual patient engagement on glycemic control in type 2 diabetes: a retrospective observational study from GluCare hybrid care model
Source: Front Endocrinol (Lausanne). 2025 Nov 20;16:1695381. doi: 10.3389/fendo.2025.1695381 (PMC12675252; doi:10.3389/fendo.2025.1695381)
Supplement: Supplementary file 1 [file Table1.docx]

Supplementary Material

# Supplementary Material

## Supplementary Tables

In the controlled group, inbound interactions decreased over time from a median of 13 (IQR: 7–26) at 3 months to 3 (IQR: 0–9) at 12 months. Similarly, in the poorly controlled group, inbound interactions followed a comparable declining trend from 17 (IQR: 5–32) at 3 months to 2 (IQR: 0–7) at 12 months.

However, within each group, there was a noticeable trend where outbound interactions were consistently higher than inbound interactions across all time points. In the controlled group, outbound interactions started at a median of 25 (IQR: 15–38) at 3 months and declined to 8 (IQR: 3–17) at 12 months, while in the poorly controlled group, outbound interactions were initially 29 (IQR: 14–46) at 3 months and gradually reduced to 9 (IQR: 3–16) at 12 months.

**Table S1: Month wise inbound and outbound interactions among controlled and poorly controlled patients**

|  | **Controlled**  **(n =63)** | **Poorly controlled**  **(n=62)** | **P value** | **Controlled**  **(n =63)** | **Poorly controlled**  **(n=62)** | **P value** |
| --- | --- | --- | --- | --- | --- | --- |
|  | **Inbound interactions**  **Median (IQR)** | | | **Outbound interactions**  **Median (IQR)** | | |
| 3 months | 13(7,26) | 17(5,32) | 0.35 | 25(15,38) | 29(14,46) | 0.33 |
| 6 months | 5(3,11) | 5(0,15) | 0.89 | 13(11,22) | 17(8,22) | 0.87 |
| 9 months | 3(0,9) | 4(0,9) | 0.9 | 9(5,21) | 15(7,20) | 0.38 |
| 12 months | 3(0,9) | 2(0,7) | 0.59 | 8(3,17) | 9(3,16) | 0.80 |

Table S2 summarizes the proportion of patients in each glycemic control group (controlled and poorly controlled) who were prescribed GLP-1 receptor agonists and dual GIP/GLP-1 receptor agonists at baseline and after 12 months of follow-up. Minimal variation was observed in prescription rates over time in both groups. Notably, the poorly controlled group consistently had a higher proportion of patients on GIP/GLP-1 therapy compared to the controlled group at both timepoints. These data suggest limited pharmacologic changes during the study period and help contextualize the observed glycemic improvements as primarily attributable to the care model and patient engagement.

**Table S2. Proportions of Patients Prescribed GLP-1 and GIP/GLP-1 Receptor Agonists at Baseline and 12 Months**

| Group | Time Point | GLP-1 Users n (%) | GIP/GLP-1 Users n (%) |
| --- | --- | --- | --- |
| Controlled Group | Baseline | 11 (17.5%) | 23 (36.5%) |
|  | 12 Months | 8 (12.7%) | 25 (39.7%) |
| Poorly Controlled Group | Baseline | 17 (27.4%) | 34 (54.8%) |
|  | 12 Months | 9 (14.5%) | 35 (56.5%) |
